# Impact of HPV infection on oral squamous cell carcinoma

**DOI:** 10.18632/oncotarget.12501

**Published:** 2016-10-06

**Authors:** Carolin Götz, Enken Drecoll, Melanie Straub, Oliver Bissinger, Klaus-Dietrich Wolff, Andreas Kolk

**Affiliations:** ^1^ Department of Maxillofacial and Oral Surgery, Technical University Munich, 81675 München, Germany; ^2^ Department of Pathology, Technical University Munich, 81675 München, Germany

**Keywords:** oral squamous cell carcinoma, HPV, p16^ink4a^, head and neck cancer

## Abstract

**Background:**

Head and neck squamous cell carcinomas (HNSCC) are often divided by their aetiology. Noxae associated collectives are compared with the human papilloma virus (HPV)-associated group, whereas different localisations of oral (OSCC) and oropharyngeal (OPSCC) squamous cell carcinomas are mostly discussed as one single group. Our aim was to show that classification by aetiology is not appropriate for OSCC.

**Results:**

HPV DNA was detected by PCR in 7 (3.47%) patients, and we identified 12 (5.94%) positive (+) cases by p16^INK4a^ immunostaining. Only 4 (1.98%) of the p16^INK4a^+ cases were + for HPV using PCR. Our homogenous collective of OSCC allowed us to compare HPV+ and HPV negative (−) patients without creating bias for tumour localisation, age, gender or tumour stage.

**Materials and methods:**

After testing OSCC samples for HPV positivity, we compared the results of two commonly used HPV detection methods, p16^INK4a^ immunostaining and HPV DNA-related PCR, on 202 OSCC patients. HPV subtypes were determined with an HPV LCD Array Kit. Clinicopathological features of the patients were analysed, and the disease specific survival rates (DSS) for HPV+ and HPV− patients were obtained.

**Conclusions:**

p16^INK4a^ immunostaining is a not a reliable HPV detection method for OSCC. Positive p16^INK4a^ immunostaining did not agree with + results from PCR of HPV DNA. Furthermore, the influence of HPV-related oncogenic transformation in OSCC is overestimated. The significance of HPV infection remains clinically unclear, and its influence on survival rates is not relevant to OSCC cases.

## INTRODUCTION

Head and neck squamous cell carcinomas (HNSCC) are one of the most common cancers in the world and are ranked sixth for men and eleventh for women in cancer frequency [1]. Although the term HNSCC is often used to describe tumours in different locations, this is not precisely correct. It would be more accurate to subdivide carcinomas by different anatomical and clinical subtypes such as OSCC (oral squamous cell carcinoma), OPSCC (oropharyngeal squamous cell carcinoma), LSSC (laryngeal squamous cell carcinoma) as well as nasopharyngeal squamous cell carcinoma (NPSCC). This analysis focuses solely on OSCC, the largest subgroup of HNSCC next to OPSCC. Significant surgical efforts, including tumour site reconstruction using free microvascular flap techniques and excellent tumour patient aftercare, have improved the long-term quality of life in tumour patients [2]. However, despite these advances, disease-specific survival rates (DSS) are still low for OSCC. Local recurrence and locoregional second carcinomas often appear within first 5 years after the primary diagnosis of OSCC, and the 5-year survival rate of advanced OSCC is approximately 30 percent [3]. TNM and UICC stages are generally still used to describe the size of the tumour and its invasion into the lymphatic system from a topographic point of view, although recently, these systems were shown to be poor at accurately predicting an outcome [4, 5]. The latter classifications of OSCC are used for outcome prediction and decision-making concerning additional radiotherapy in cases of positive nodal metastasis [6]. Therefore, the need to detect reliable predictive biomarkers, genetic alterations or epigenetic modifications in OSCC to estimate recurrence for therapy stratification and planning is significant, and it is also important to identify significant molecular pathways to precisely select patients who are suitable for additional therapy [7–9].

**Figure 1:**
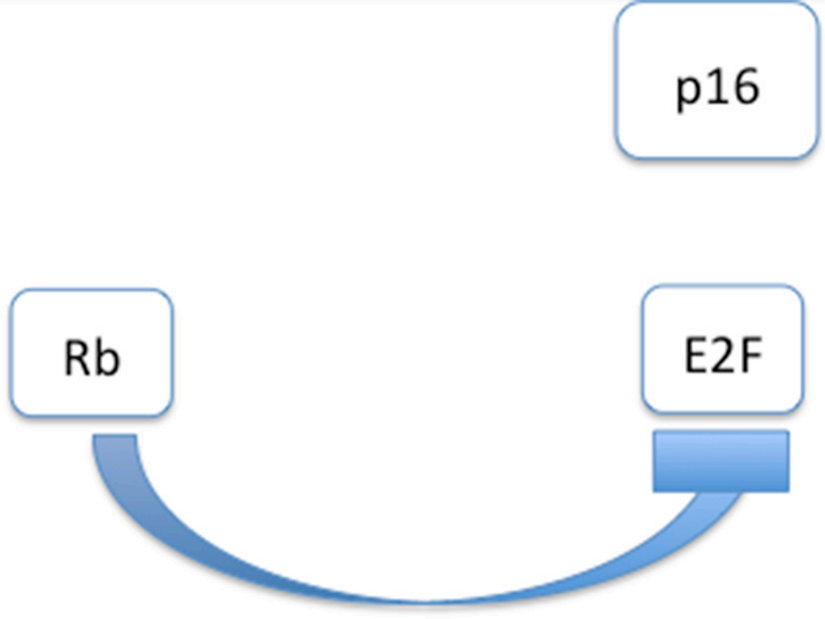
Interaction modell of p16^INK4a^ in HPV negative cases: Tumoursuppressor Retinoblastoma gene (Rb) is inhibiting transcription factor E2F As a consequence, p16^INK4a^ is not affectd by E2F.

**Figure 2:**
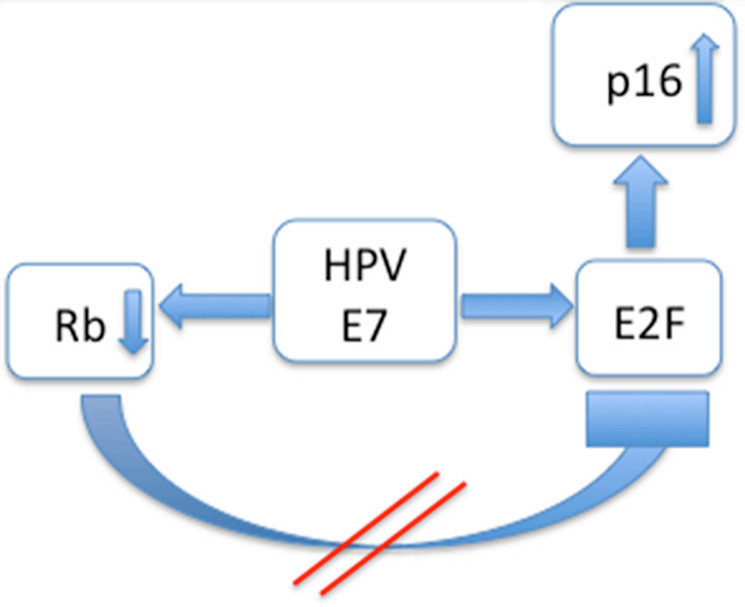
Interaction modell of p16^INK4a^ in HPV positive cases: The HPV associated oncoprotein E7 acts in a double way: Rb is inhibited and E2F is promoted As a result E2F is upregulated and p16^INK4a^ is overexpressed.

Recently, HPV has been discussed as a relevant predictive biomarker for OSCC [10, 11]. Higher rates of HPV+ tumours, better outcomes for HPV+ patients, and improved survival rates compared to HPV− groups have often been published in studies that did not differentiate between OSCC and OPSCC [12, 13] and did not examine other confounders significantly influencing DSS. Only few studies were published which differentiate sufficiently between the anatomical localisations [14]. In OPSCC in particular, lymphoid tissue and lymphoid precursor cells interact with HPV infection and also carcinoma cells through various molecular mechanisms [15]. Because of this, HPV exhibiting p16^INK4a^ positivity is more often seen in OPSCC [16], requiring OPSCC to be judged separately from OSCC. HPV infection is often detected with immunohistochemistry (IHC) of p16^INK4a^. HPV associated oncoproteins as E7 are influencing the expression of p16^INK4a^ (Figures 1 and 2) [17–19]. Therefore p16^INK4a^ is used as an HPV-related surrogate marker in many study protocols because the procedure is well-established and time saving [20, 21]. It has a distinct role in cell cycle regulation [22], acting as a tumour suppressor protein. It is well known that detection of HPV infection with p16^INK4a^ staining often does not give accurate results [23, 24]. Another pitfall making survival comparisons even more difficult is the comparison of non-homogeneous groups. Bias by age and corresponding comorbidities as well as different tumour stages influence survival rates. Often, false conclusions are drawn based on different survival rates without regarding the diverse ages of the HPV+ and −groups.

To date, only a few OSCC studies have examined homogenous groups in terms of patient age and tumour stage. The aim of the current analysis was I) to compare a completely homogenous group and assess two of the most commonly used HPV detection methods with respect to HPV expression rates and II) to evaluate HPV status for relevance to patient survival and clinical outcome.

## RESULTS

### p16^INK4a^

**Figure 3:**
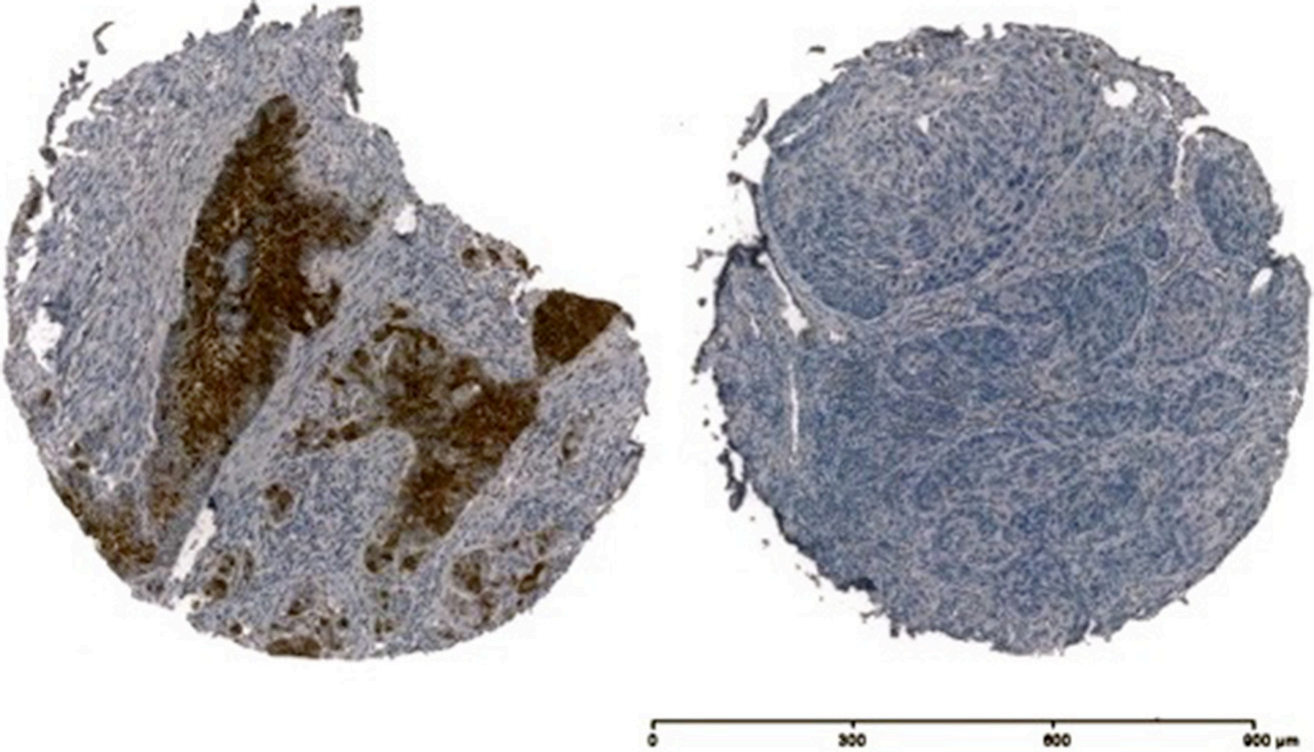
Immunohistochemistry of p16^INK4a^ of two OSCC samples, on the left side p16^INK4a^ + and on the right side p16^INK4a^

12 (5.94%) patients tested positive for p16^INK4a^ (Table 1). These results were clear with a strong intensity staining in all 12 cases (Figure 3), and cells with no detectable staining were considered negative (Figure 3). No intermediate staining was evaluated. All IHC results were evaluated under a light microscope (Zeiss, Jena, Germany). Samples of HPV+ and − cervical cancer were used as positive and negative controls. No difference of p16^INK4a^ staining was given by the different tumour localisations as the centre of the tumour, the invasion front and the lymph nodes. If positivity/negativity of p16^INK4a^ was given, every localisation was positive/negative for p16^INK4a^.

**Table 1:** Clinicopathological features

Clinical Parameters	HPV − * (*n* = 195)	HPV + * (*n* = 7)
Median age in years (range)	57.6 (29.5–85.8)	57.4 (44.3–65.8)
Gender		
Male/Female	141/54	4/3
UICC Stage		
I	48	×
II	35	×
III	38	3
IVa	74	4
Tumor Size		
T1	72	2
T2	73	1
T3	21	2
T4a/b	29	2
N Stage		
N0	100	3
N1	35	2
N2	60	2
Extracapsular spread	21	2
Grading		
G1	12	×
G2	130	5
G3	2	2
p16		
Positive	8	4
Negative	187	3

## HPV DNA PCR

7 (3.47%) patients were scored as HPV+ based on the PCR results (Figure 4.1). A sample from an HPV+ cervical carcinoma was used as a positive control (+C) to confirm HPV infection. Control PCR (Figure 4.2) was performed in every case to ensure sufficient amounts of DNA. Only in 4 (1.98%) cases did the PCR result correspond to positive p16^INK4a^ staining. In 8 (3.96%) cases, p16^INK4a^ staining was positive, but the PCR result was negative. In 3 (1.49%) cases, p16^INK4a^ staining was negative but the PCR for HPV was positive. In conclusion, p16^INK4a^ staining for the detection of HPV is insufficiently sensitive and specific.

**Figure 4:**
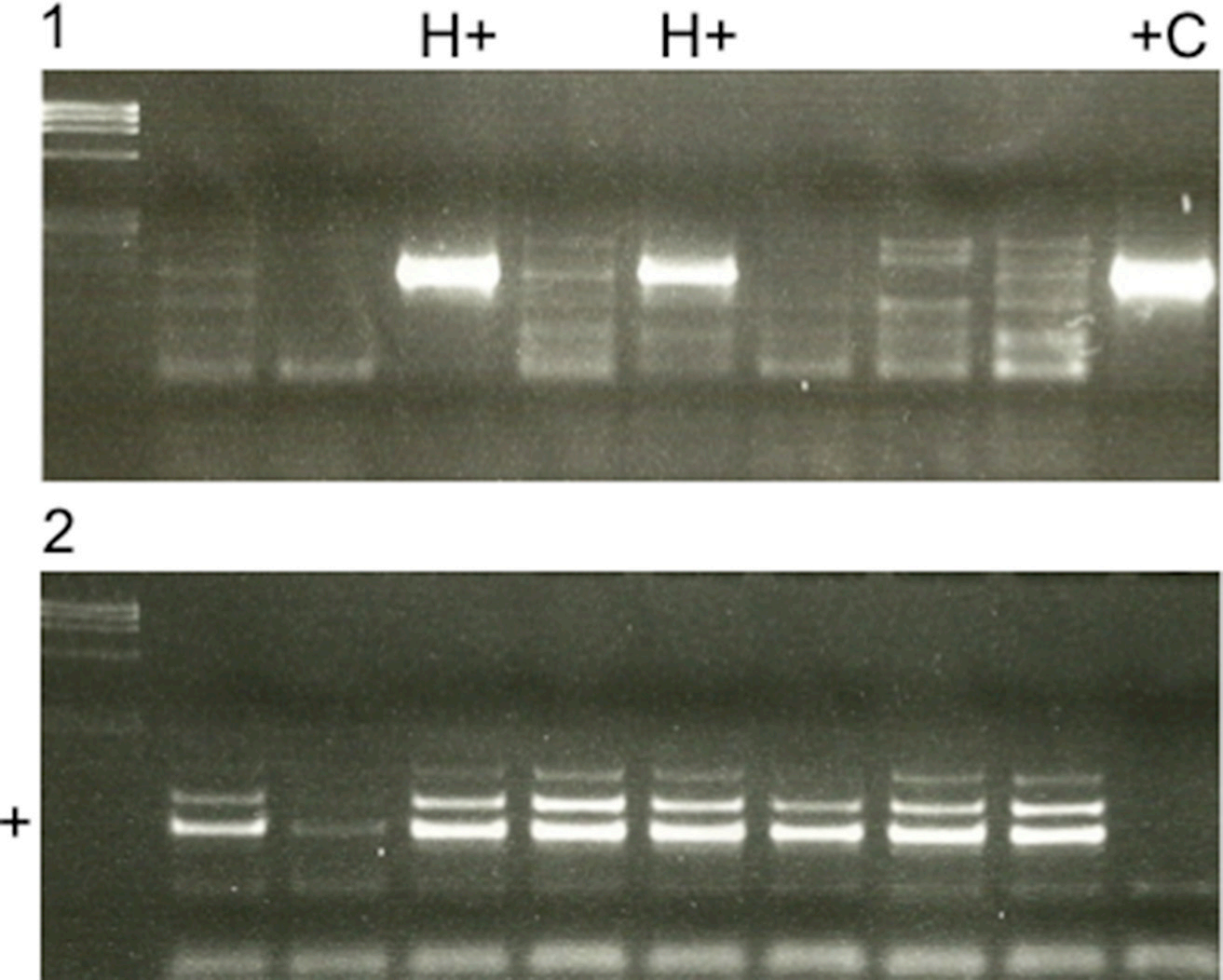
(**1**) PCR results for detecting HPV positive (H+) and negative cases (H−). A HPV positive cervix carcinoma was used as a positive control (+C). (**2**) A control PCR was performed in every case to detect sufficient amounts of DNA. If DNA amounts were insufficient as shown in row 2, DNA was newly extracted from FFPE. Otherwise a clear signal was seen in the specific row (+).

### Detection of HPV subtypes

To identify the HPV subtype, each patient was scored with the HPV LCD Array Kit (Zytomed Systems, Berlin, Germany). Furthermore, it served as proof of the reliability of the PCR method, as there were no discordant results between the PCR compared to the HPV LCD Array Kit, indicating that every OSCC identified as HPV+ by PCR was also positive with the LCD kit. In 5 OSCC cases, HPV subtype 16 was present, in one case HPV subtypes 16 and 52 were present, and in one case HPV subtypes 16 and 53 were present.

### Clinical data

Relevant clinical data from the 202 included patients with an OSCC diagnosis are listed in Table 1. Risk factors such as smoking and alcohol consumption were evaluated in comparable equal numbers from both groups. The majority of both sub-groups smoked, and the combination of smoking and regular alcohol consumption was observed in 10 percent of both sub-groups. Karnofsky performance status was assessed for every tumor patient. All patients of the current study had a Karnofsky status between 90–100 per cent, independent of HPV positivity. Due to high Karnofsky stages, the status was not significantly associated with the survival rates.

The survival rates from both groups were not significantly different according to univariate and multivariate analyses (*p >* 0.05). HPV+ patients with OSCC had an average overall survival (OS) of 33.17 months (sd 5.21; 95% Confidence Interval (CI): 22.96−43.38), and HPV− patients showed an average OS of 78.34 months (sd 4.27; 95% CI: 69.99–86.70).

Survival rates were also not significantly different between p16^INK4a^+ cases (average OS 40.08 months, sd 4.28; 95% CI: 69.99−86.77) and p16 ^INK4a^**−** cases (average OS 78.38 months, sd 6.55; 95% CI: 27.24−52.92). HPV+ / p16^INK4a^+ cases had also no better survival rates (average OS 31.56 months, sd 16.62; 95% CI: 14,94−48.18).

Both local recurrence and lymph node recurrence played a major role in survival (*p* = 0.001) and was evaluated to the same degree in 52 patients. Three of these patients were HPV+, four were positive for p16^INK4a^, and two of the p16^INK4a^+ patients were also HPV+.

Recurrence free survival (RFS) of HPV+ patients was an average of 8.56 months (sd 1.56; 95% CI: 5.51−11.61); HPV− patients showed an average RFS of 18.66 months (sd 2.57; 95% CI: 13.63–23.69).

Recurrence free survival (RFS) of p16^INK4a^+ patients was an average of 22.05 months (sd 5.78; 95% CI: 10.73−33.38), and p16^INK4a^- patients had an average RFS of 17.70 months (sd 2.54; 95% CI: 12.73−22.67).

The UICC stage (*p* = 0.031), patients' ages (*p* = 0.012) and lymph node metastasis (*p* = 0.003) at the time of primary diagnosis had a significant influence on overall survival rates independent of HPV status or p16^INK4a^.

In contrast, patient gender, T category, extra capsular spread and tumour grading were not significantly associated with overall tumour related survival or DSS (*p >* 0.05) independent of HPV or p16^INK4a^ status. Resection margins were assessed and were R0, tumour free, in every case.

## DISCUSSION

There is an ongoing discussion about the impact of an HPV infection on the prognosis and therapy regimes for HNSCC. To date, there are many ambiguities in the field. Because of unknowns in the literature, this study was performed to evaluate the HPV infection rate in a large homogenous collection of OSCC patients, examining different HPV detection methods and patients' overall and recurrence free survival. To our knowledge, this is the first large study that does not show HPV+ status improves the survival rates of OSCC patients. Furthermore, this study demonstrated that HPV infection only occurs in a relatively small number of OSCC cases. In the literature, the rate of HPV positivity in HNSCC is provided with a wide range and often substantially differs between from one study to another [32, 33]. Further examination of published data shows that the wide range of HPV+ status in SCC is the result of poor differentiation between OSCC and OPSCC [34, 35]. Studies that only included OSCC in their evaluation show a comparably smaller number of HPV+ SCC, similar to this study [36, 37]. However, studies focusing on OPSCC, especially tonsil SCC, have higher HPV positivity rates [38] because the virus interacts with lymphoid tissue. The HPV has a selective tropism for the epithelium lining the tonsillar crypts. This interaction makes the difference to sites where lymphoid tissue is not the dominant tissue, such as the oral cavity. Some similarities in morphology and function are given in OPSCC and nasopharyngeal squamous cell carcinoma (NPSCC). The numbers of HPV positivity in NPSCC have also a big variety and are depending of the collectives and detection methods. Some experts, also written in the guidelines from the College of American Pathologists, promote HPV testing in EBV-negative NPSCC [39]. Further studies could give helpful knowlegde for patients with HPV+ NPSCC. A topic of interest for further research is the interaction between HPV and different tissue types, especially lymphoid tissue, in OPSCC [40]. Better outcomes of HPV+ HNSCC are mostly described for HPV+ OPSCC regarding adjuvant therapy strategies as adjuvant radiation [41] or antibody specific therapy [42].

Evaluation of the HPV detection methods that have been used in several studies provides an additional explanation for the wide range of HPV+ SCC reported. Various methods are available for HPV detection. As often discussed, an insufficient method for HPV detection that is still often applied is p16^INK4a^ immunostaining [43]. Because this protein interacts with the cell cycle in multiple ways, it is an insensitive predictor of HPV status with a low predictive value for HPV infection [24] and should be viewed as an independent marker influencing the cell cycle of carcinoma cells. This supports our findings that p16^INK4a^ immunostaining and overexpression is an insufficient HPV detection method, because we observed a high failure rate in cases that were positive for p16^INK4a^ but were actually HPV−. Using the latter method, the number of false-positive HPV+ patients would be 50 percent higher. Studies that assessed p16^INK4a^ immunostaining without other detection methods have a higher positive rate of HPV infection [44]. Remarkably, a strong correlation between p16^INK4a^ overexpression and OPSCC, mostly tonsil SCC, is seen [45]. However, in OPSCC as well as in OSCC, the correlation between p16^INK4a^ overexpression and HPV requires further research.

We used RT-PCR of extracted DNA from the FFPE of OSCC as the standard method for HPV detection and collected valuable results, as described previously [44]. One advantage is the possibility to use FFPE for RT-PCR, because this allowed a retrospective investigation of a large group OSCC patients as a screening method. Additionally, the HPV subtypes were detected with the LCD Array using DNA extracted from FFPE. The use of RT-PCR and the latter LCD array gives more advantages as relaiable results and better economical aspects than the use of the Sanger sequence analysis [46]. With the additional method of the LCD Array, we obtained more information about the oncogenic risk potential of underlying HPV subtypes in addition to confirmation of the RT-PCR results, as a positive result for the LCD Array was only observed if the OSCC DNA was HPV+. HPV subtype 16 was detected in all HPV+ cases, as the most common oncogenic HPV subtype described for OSCC in literature before [47] promote better OS rates. Further evaluated subtypes were 52 and 53, the latter one is ranked as potentially oncogenic. These results show the relevance of HPV driven carcinogenesis in the HPV+ cases of the current study. False positive results for HPV infection and an overestimation of the number of HPV+ patients could therefore be excluded in view of the results presented here. The low incidence of HPV in OSCC patients in the literature was clearly confirmed with the results of this study. p16^INK4a^ overexpression was not relevant to differences in age, gender, tumour stage or the survival rates of p16^INK4a^*+* patients, suggesting that p16^INK4a^ plays a less important role in OSCC. Recent studies focusing on p16^INK4a^ expression have described improved survival rates in p16^INK4a^*+* patients without differentiating between the two main localisation subgroups of OSCC and OPSCC. Only a few OSCC studies focused on p16^INK4a^, making a comparison of the literature with this data difficult. Tumour patients treated over a certain period of time were collected, and homogeneous groups of tumour patients are sometimes rare but are a strong requirement for any serious comparison. Our study group showed relatively homogenous characteristics since both in the HPV+ and HPV− collective advanced tumor stages were occuring in high numbers/percentage of the total amount of included HPV+ and HPV− patients and allowed comparisons between HPV+ and − without bias. In the literature, HPV+ patients are primarily younger, which could certainly influence OS and DSS and promote better OS rates [48]. Our collective also showed homogenous results for all of the evaluated variables (Table 1). The patient specific data was completely independent of the presence of an HPV infection. The limitations of this study are, as discussed before, the lack of knowledge about the specific molecular pathways of p16^INK4a^ and HPV. Further, multiple ways of interactions of HPV and different types of tissues of the oral cavity and pharynx could yet not be explained sufficiently. Another aim for prospect studies could be the distinct evaluation of these interactions.

## MATERIALS AND METHODS

### Patients

Two hundred two patients treated between 2009 and 2011 at our maxillofacial surgery department with the intention of curative treatment were included in our study. The inclusion criteria were the availability of relevant data (Table 1) from patients diagnosed with OSCC for statistical evaluation and of formalin fixed and paraffin embedded tissue (FFPE) for laboratory use. Follow up data were available for every patient of the study. All patients attended regular follow up examinations at our departement. Tumours of the tongue base, and tumours with involvement of the latter anatomical side were excluded in the current study to avoid bias of HPV interacting with lymphoid tissue. To the present knowledge these tumours should be classified as OPSCC. The therapy regimes of the patients included were primary surgery, with intraoperative margin control with the help of frozen sections and with neck dissection. Due to the german guidelines of oral cancer surgery [25] all patients underwent an ipsilateral selective neck dissection with clearance of the nodes of the ispsilateral levels I-III. In case of metastatic nodal disease (pN1 or pN2) that was intraoperatively confirmed by frozen section, a modified neck dissection with extended nodal clearance down to level IV and V and contra lateral neck dissection of level I-III was done. Postoperative adjuvant cisplatin-based chemoradiation, with a dose depending from 50 to 66 Gy, was performed in case of pN1, pN2 or tumor infiltration of the jaw or locally infiltrating tumor growth of the oral cavity (T4a/b), positive microscopic resection margins and/or extracapsular spread, according to the German guidelines for oral cancer.

Exclusion criteria were death resulting from a cause other than OSCC, distant metastasis at primary diagnosis and the use of primary radiochemotherapy before operation. The methods were approved by the local ethics committee (no. 212108) and are in accordance with the Declaration of Helsinki. All patients gave their consent.

### Tissue microarray construction

Two independent pathologists defined the centre of the tumour and the invasion front and the lymph nodes of every study patient. The tissue was formalin fixed and paraffin embedded in blocks. The pathologists then marked the areas to be represented in the tissue microarray (TMA). A minimum of two tumour cores from the centre of the tumour, the invasion front and the corresponding lymph nodes with a 6 mm core size were assembled into the TMA using a Tissue Microarrayer (Beecher Instruments, Sun Praierie, USA) as described before [26].

### IHC of p16^INK4a^

Four-micron TMA sections were stained with p16^INK4a^ antibody (CIN tec; Ventana Medical Systems, Tucson, AZ, USA) according to the manufacturer's recommendations. Visualisation of staining was performed with the chromogenic substrate 3.3′-diaminobenzidine (DakoCytomation; 10 min, room temperature). Finally, nuclei were counterstained with Mayer's acid haematoxylin, and the slides were covered with Pertex mounting medium (MEDITE, Burgdorf, Germany) as described previously [27]. Positivity of p16^INK4a^ was evaluated as described before with a methodological standardization to ensure result reproducibility [28].

### DNA detection with PCR

FFPE full-face slides of the central tumour area from every patient were cut in 15-micron sections to extract DNA [29, 30]. Extraction of HPV DNA was performed with a QIAamp DNA FFPE Tissue Kit (Qiagen GmbH, Hilden Germany), and PCR was performed with prime Mix A and Mix B (Mix My 11/09 and Mix 125) provided by the LCD-Array HPV 3.5 kit (Zytomed Systems, Berlin, Germany).

### Detection of HPV subtypes

After exhibiting a positive HPV signal by PCR, the HPV 3.5 LCD Array Kit (Zytomed Systems, Berlin, Germany) was used to distinguish which of the 32 HPV subtypes were present, including high and low risk types, as described previously [31]. After amplification of HPV DNA, hybridization was performed on the DNA LCD Array chip. Specific HPV+ probes were immobilised on the LCD chip surface. After washing the chips and drying via centrifugation, the visualisation of bound amplicons was possible with an enzyme-substrate reaction. Stained arrays were analysed with a scanning device and software.

### Statistics

Data and figures were analysed with the ‘Statistical Package for the Social Sciences’ (SPSS for Windows, release 22.0.0, 2013, SPSS Inc., Chicago, IL, USA). Cox regression was used for survival analysis. Categories were tested for associations using cross tabs (Chi-squared-test). To compare groups, the non-parametric Mann Whitney *U-test* was used. Probabilities of less than 0.05 were considered significant.

## CONCLUSIONS

p16^INK4a^ immunostaining should not be used for HPV detection in OSCC. Furthermore, OSCC as a distinct localisation with principally squamous cell tissue should be clearly differentiated from OPSCC, which mainly contains lymphoid tissue. Considering the reliability of current data from the literature and the results presented here, there is no justification to promote therapy de-escalation in OSCC cases based on an HPV infection. HPV infection in OSCC is of low incidence and does not seem to be a predictive biomarker.
